# Prevalence of sleep disturbance among adolescents with substance use: a systematic review and meta-analysis

**DOI:** 10.1186/s13034-023-00644-5

**Published:** 2023-08-26

**Authors:** Doreen Phiri, Vivi Leona Amelia, Muhammad Muslih, Lindelwa Portia Dlamini, Min-Huey Chung, Pi-Chen Chang

**Affiliations:** 1 School of Nursing, College of Nursing, Taipei Medical University, Taipei City, Taiwan; 2 Universitas Muhammadiyah Purwokerto, Purwokerto, Central Java Indonesia; 3 School of Nursing, Faculty of Health Science, University of Muhammadiyah Malang, Malang, Indonesia; 4 Department of Nursing, Taipei Medical University-Shuang Ho Hospital, New Taipei City, Taiwan

**Keywords:** Adolescents, Substance use, Sleep disturbance, Meta-analysis

## Abstract

**Purpose:**

Sleep disturbance has become a major challenge among adolescents worldwide. Substance use is among the most common factors contributing to sleep disturbance. This systematic review and meta-analysis examined the prevalence and categories of sleep disturbance among adolescents with substance use.

**Methods:**

We comprehensively searched for relevant studies published in the following databases from inception to August 2022: CINHAL (via EBSCOhost), PubMed, Scopus, Ovid Medline, Embase, ProQuest, and Web of Science. Data analysis was performed using Comprehensive Meta-Analysis version 3 software. We used a random-effects model to pool prevalence rates with 95% confidence intervals (CIs). Forest plots and *p* values for the Cochran Q statistic were used to evaluate heterogeneity among studies. Subgroup and meta-regression analyses were performed to compare the groups and identify the sources of heterogeneity.

**Results:**

We examined 18 studies that reported insomnia, hypersomnolence, sleep-related breathing disorders as sleep disturbances among adolescents with the use of alcohol, smoking, marijuana, and coffee. The total sample was 124,554. The overall prevalence rate of sleep disturbance was 29% (95% CI: 0.201–0.403). Subgroup analysis revealed that the prevalence rates of insomnia and hypersomnolence were higher among alcohol users (31%; 95% CI: 0.100–0.654) and smokers (46%; 95% CI: 0.232–0.700). The study design and method of assessment groups were the significant moderators that showed the source of variation in the included studies.

**Conclusion:**

Sleep disturbance is highly prevalent among adolescents with substance use. Insomnia and hypersomnolence are more prevalent among alcohol users and smokers, respectively. On the basis of our findings, health-care providers can develop effective targeted interventions to reduce substance use, prevent sleep disturbance, and promote healthy sleep habits among adolescents.

**Supplementary Information:**

The online version contains supplementary material available at 10.1186/s13034-023-00644-5.

## Introduction

Sleep disturbances have become a public health concern worldwide. Insomnia, sleep-related breathing disorders (SRBDs), and hypersomnolence are the most common categories of sleep disturbance in the *International Classification of Sleep Disorders-Third Edition* (*ICSD-3*) [1]. Insomnia is defined as difficulty initiating or maintaining sleep, resulting in poor sleep quality that impairs the daytime functioning of an individual [2]. Symptoms of hypersomnolence include excessive daytime sleepiness (EDS), difficulty waking up in the morning, and not feeling refreshed despite having adequate sleep [3]. SRBDs include obstructive sleep apnea (OSA), central sleep apnea, sleep-related hypoventilation disorders, and sleep-related hypoxemia disorders [4]. OSA is the most common SRBD, and its symptoms include snoring and difficulty breathing when sleeping (DBS) [5, 6]. Globally, numerous individuals, including adolescents, experience sleep disturbances [7]. Adolescents are among the age groups highly affected by sleep disturbance [8, 9]. Adolescents, defined as those aged between 10 and 24 years [10], experience a unique phase of physical, psychosocial, and cognitive development [11]. In adolescents, healthy sleep promotes growth, learning, and cognitive development and is also beneficial for their physical and mental well-being [12, 13]. Research has shown that the circadian rhythm system and sleep homeostatic system, which are the neurobiological processes that occur during adolescence, result in significant changes in adolescents’ sleep patterns [14, 15]. As the adolescent period progresses, the circadian clock shifts to a later time, resulting in sleep phase delays [15]. In addition, due to a gradual increase in sleep pressure during sleep homeostasis, late adolescents experience a delay in bedtime [15, 16]. This change causes adolescents to experience insufficient sleep, late bedtimes, and possibly poor sleep quality, which progress into insomnia, delayed sleep phase disorders, and daytime sleepiness [17–19]. According to the literature, sleep disturbance is associated with poor mental health status, poor academic performance, aggressive behavior, and suicide among adolescents [20–24]. A previous review reported that the prevalence of sleep disturbance among adolescents in China was 28%, ranging from 8 to 54.7% [8]. Another study reported that 14–33% of adolescents experience sleep problems in Western countries [25]. Furthermore, the prevalence rates of insomnia and EDS were 16.1% and 17%, respectively, among adolescents in China [26]. The aforementioned findings imply that sleep disturbance among adolescents is a severe concern that warrants attention and further research.

In adolescents, sleep disturbances can be caused by substance use [27, 28]. Substance use is among the top five causes of sleep disturbance among adolescents [29, 30]. Tobacco, cigarettes, alcohol, caffeine in coffee, and marijuana are the most common substances used by adolescents [31]. Previous studies have shown that prevalence rates of substance use among adolescents in Western countries range from 22 to 40% [32–35]. About 15% of adolescents aged 18 years were reported to consume alcohol [36]. In addition, 36%, 39.8%, and 33% of adolescents were reported to be smokers in Canada, England, and the United States, respectively [37]. Generally, most substances are known to exert a direct effect on sleep architecture and neurotransmitter regulation [38]. According to the literature, alcohol impairs the rapid eye movement phase of sleep, thereby affecting sleep duration and continuity, and additionally, it can induce drowsiness [39]. Despite the fact that studies have found a bidirectional relationship between alcohol use and sleep disturbances, the adverse outcomes of alcohol on sleep among adolescents are prominent [40]. Furthermore, nicotine, which is found in smoking substances such as tobacco and cigarettes, is suggested to be a strong stimulant of neurotransmitters affecting the sleep-wake cycle, neuromuscular activity of the upper airway, inflammation of the airway, and the arousal mechanism [41]. The neurological changes may result in adolescents having trouble getting sleep, falling asleep, having difficulties breathing during sleep, and having daytime sleepiness [41]. Other substances like caffeine in coffee and marijuana have been reported to be widely used for the purpose of trying to increase alertness, enhance academic performance, gain confidence, and release tension among school-going adolescents [42–44]. Caffeine is a stimulant responsible for the release of catecholamines that affect an individual’s emotional, cognitive, and motor functionality [45]. It has been recommended that adolescents limit their intake of caffeine to 300 mg per day [46]. Research has shown that adolescents with high coffee consumption are more likely to feel tired in the morning and have trouble sleeping [47]. Regarding marijuana use, also known as cannabis, previous studies have demonstrated that it contains Δ9-Tetrahydrocannabinol (Δ9-THC) and Cannabidiol (CBD) that affect the sleep structure and further alter the development of neurons in the brain of adolescents [48, 49]. About 6.5% of adolescents in the United States of America reported the use of marijuana in 2018, while 37% of high school adolescents used marijuana in 2019 [50, 51]. Moreover, 3.7% of adolescents were regular users of marijuana in the United Kingdom [52]. Additionally, 80% of adolescents were estimated to use marijuana in New Zealand [53]. Generally, smoking, alcohol, coffee consumption, and marijuana use can cause headaches, irritability, and hallucinations that may disturb normal sleep [28]. Compared with adolescents who do not use substances, those who use substances experience more sleep disturbance [27]. Sleep disturbance among adolescents with alcohol, smoking, coffee, and marijuana should be evaluated to determine effective measures for promoting healthy sleep among adolescents.

Although sleep disturbance is a major health concern among adolescents, most previous studies focused on the adult population and investigated only one or two categories of sleep disturbance among individuals. For instance, a study examining adult alcohol users reported that 90% and 60% of them experienced EDS and insomnia, respectively [28]. In addition, a previous study reported that 30% of marijuana users experienced sleep disturbances [54]. Most of the previous studies did not include coffee, although it is among the most commonly used substances among school-going adolescents [27]. Furthermore, the setting of the studies differed because the majority of them were conducted in Western countries. Moreover, due to methodological differences such as designs, sample size, measurement, and conceptualization of sleep disturbances, the results of previous studies are inconsistent. To our knowledge, no meta-analysis has examined the prevalence of sleep disturbance in adolescents with substance use. Therefore, this systematic review and meta-analysis investigated the prevalence of sleep disturbances, including insomnia, hypersomnolence and sleep-related breathing disorders, among adolescents with alcohol, smoking, coffee, and marijuana.

## Methods

This study was conducted in accordance with the Preferred Reporting Items for Systematic Review and Meta-Analysis [55] and is registered in PROSPERO (registration number: CRD42021266832).

### Literature search

We selected search terms following the population, exposure, and outcome (PEO) format, where P is adolescents, E is substance use, and O is sleep disturbance. We systematically searched for relevant studies in CINHAL (via EBSCOhost), PubMed, Scopus, Ovid Medline, Embase, PsychINFO (via EBSCOhost), and the Web of Science from inception to October 2021. Furthermore, we identified additional relevant studies by manually searching the reference lists of the included studies and other reviews. Endnote version X9 was used to screen papers. Supplementary Table S1 presents the complete details of the search strategy.

### Inclusion and exclusion criteria

We included observational studies (cross-sectional or cohort) involving adolescents aged 10 to 24 years [10] who used substances (alcohol, smoking, marijuana, and coffee) and experienced sleep disturbance due to substance use (insomnia, hypersomnolence, and SRBDs). We excluded research abstracts, duplicate studies, low-quality studies, non-observational studies, and non-English studies.

### Screening and selection of relevant studies

Two independent reviewers (D.P and M.M) screened and selected the articles. A third reviewer (V.L.A) was consulted where the reviewers could not agree. We used Endnote Software version X9 to screen studies. First, we removed duplicates and evaluated the title and abstract of the identified studies. We excluded unrelated and irrelevant (e.g., those focusing on other age groups, other substances, or other sleep disturbance categories) studies and screened the full text of studies. Following our inclusion and exclusion criteria, we included only relevant full-text studies.

### Data extraction

Two independent reviewers (D.P. and M.M.) extracted the following information: (1) study characteristics (publication year, country in which the study was conducted, study design, study setting, sample size), (2) population characteristics (demographics and type of substance use, namely alcohol, coffee, smoking, and marijuana), and (3) outcome measures (sleep disturbance measures, method of assessment, and the overall prevalence rate of the categories of sleep disturbance, namely insomnia, hypersomnolence, and SRBDs with 95% CIs). Disagreement between the two reviewers was resolved through consultation with a third reviewer (V.L.A.) [56].

### Quality assessment

Two reviewers (D.P. and M.M.) reviewed the methodological study’s quality and potential bias. Each study was appraised using a critical appraisal tool developed by the Joanna and Briggs Institute (JBI) [57, 58]. This tool consisted of nine questions, and their responses were either yes, no, unclear, or not applicable. The number of positive answers for each question was determined to examine the risk of bias and the methodological quality of studies. Each “yes” represented a score of 1, and “no,” “unclear,” and “not applicable” represented a score of zero. Based on the JBI appraisal tool scores, percentages were calculated, and studies with scores of ≤ 49%, 50–60%, and ≥ 70% were classified as having a high, moderate, or low risk of bias. High risk of bias denoted a low quality, moderate risk of bias donated moderate quality, and low risk of bias denoted a high risk of bias, respectively [59].

### Data analysis

We used Comprehensive Meta-Analysis software version 3 [60] to calculate the pooled prevalence rates with 95% CIs. Based on the study characteristics, the included studies were not homogenous. We, therefore, used a random-effects model to consider the uncertainty caused by the variations among the included studies [61]. Forest plots and *p* values for the Cochran Q statistic were determined to evaluate the heterogeneity of studies. A *p* value of < 0.05 indicated significant heterogeneity. To determine the proportion of heterogeneity, *I*^2^ values were calculated. An *I*^2^ value of 0% indicated the absence of heterogeneity, and those of 1–25%, 25–75%, and > 75% indicated low, moderate, and high heterogeneity, respectively [62]. To examine the prevalence rate among the categories of sleep disturbances, we performed a subgroup analysis in which we grouped each category by the different types of the substances used by adolescents. To identify the source of heterogeneity, subgroup and meta-regression analyses were performed. The following categorical variables were included in the subgroup analysis: geographic location, study setting, assessment method, instrument used, and study design. Age and sample size were included in the meta-regression analysis. Furthermore, we performed a sensitivity analysis to determine the stability of the results [63]. To perform sensitivity analysis, we used the leave-one-study-out technique.

### Publication bias

We generated a funnel plot to examine the presence of publication bias [64]. Statistical evaluation was performed using Begg and Mazumder rank correlation and Egger’s tests [65]. Begg and Mazumder rank correlation involves the evaluation of the relationship between the ranks of effect sizes and the ranks of their sampling variations [65]. Egger’s test provides the degree to which a funnel plot is asymmetric based on the measurements of the intercept from the regression of the standard normal deviations against precision [65, 66]. A *p* value of < 0.05 denoted the presence of statistically significant publication bias. Adjusted pooled prevalence rates were calculated using Duval and Twidees’s trim and fill test with consideration of missing studies [67].

## Results

### Inclusion of studies

**Fig. 1:**
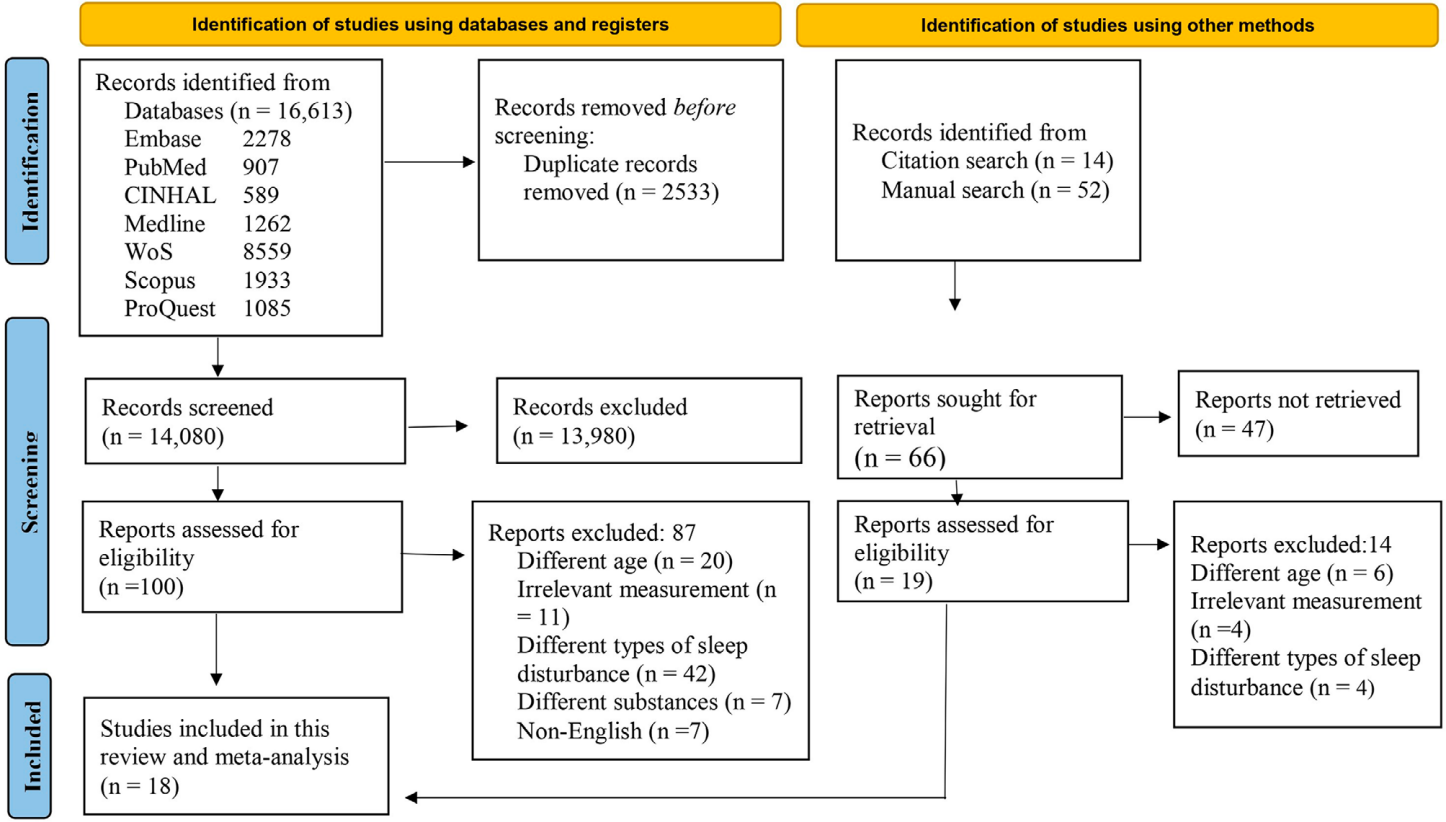
PRISMA Flowchart

Figure 1 presents the PRISMA flowchart for study screening and selection. We used search strings to obtain articles in different databases: For instance, the following combinations were used in Embase database; (Teenage* OR adolescent):ti,ab,kw,de OR “Adolescence”/exp OR “teenager”/exp AND (“Substance use” OR “alcohol use” OR “marijuana use” OR “tobacco smoking” OR “cannabis use” OR “coffee use” OR “cigarette smoking”):ti,ab,kw,de OR “Substance-related disorders”/exp OR “alcohol drinking”/exp OR “marijuana use”/exp OR “tobacco smoking”/exp OR cannabis/exp OR coffee OR “cigarette smoking”/exp AND (“Sleep disturbances” OR insomnia OR “inadequate sleep” OR sleeplessness OR Sleep OR hypersomnolence OR “Sleep problem” OR “sleep-related breathing disorders” OR “insufficient sleep” OR):ti,ab,kw,de AND “Sleep initiation and maintenance disorders”/exp OR “disorders of excessive somnolence”/exp OR “sleep apnea syndromes.“ The electronic database search yielded 16,613 articles. Of those, 2533 duplicate articles were removed. After screening the abstracts and titles of the remaining 14,080 articles, we excluded 13,980 articles. We evaluated the full text of the remaining 100 articles. Among those, we excluded 20 studies that enrolled individuals belonging to different age groups, 42 studies that included patients with different types of sleep disturbance, 11 studies using irrelevant measurements, 7 studies examining different substances, and 7 studies that were not published in English. The remaining 13 studies were included in the meta-analysis. We identified an additional 66 studies after manually searching the reference lists of the relevant studies. After screening, we excluded 47 articles and further screened the remaining 19 articles. We excluded six studies that enrolled individuals belonging to different age groups, four studies using irrelevant measurements, and four studies that included patients with different types of sleep disturbance. The remaining five articles were included in the analysis. Finally, 18 articles were included in this study (Supplementary Table S2).

### Characteristics of included studies

**Fig. 2:**
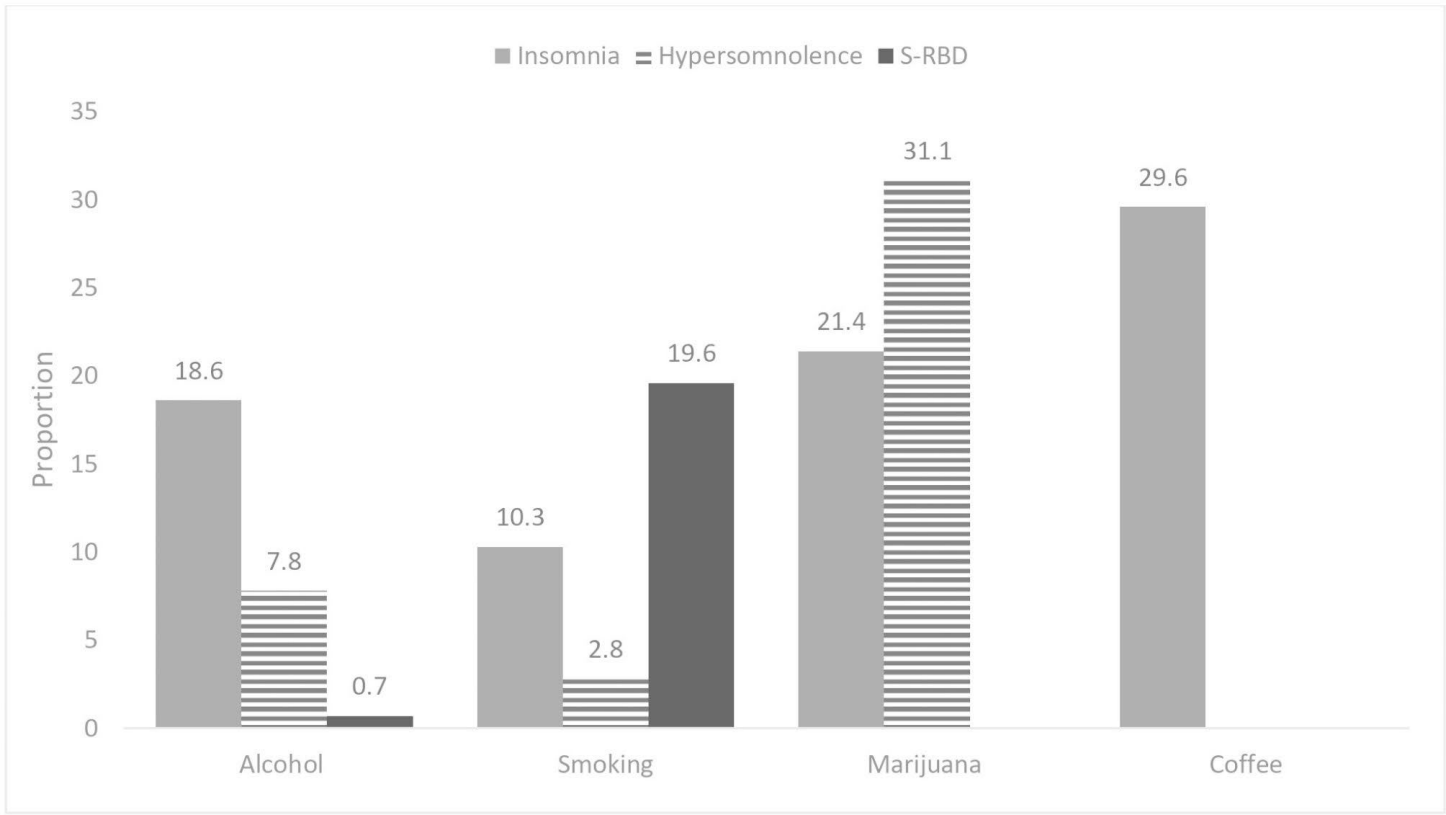
Proportions of included studies in relation with sleep disturbance and substance use

Table 1 summarizes the characteristics of the included studies. Supplementary Table S3 provides the complete details of all the included studies. Eighteen articles published between 1993 and 2021 were included in this study [68–85]. All the studies included both female and male adolescents with a mean age of 15 (standard deviation: 3.32, range: 10 to 20) years. The percentage of males was slightly higher than that of females (50.2% vs. 49.8%). The total sample size was 124,554, ranging from 596 to 28,839. The majority of the studies were conducted in the United States (n = 6) and Europe (n = 6), and five studies were conducted in Asia and one in Oceania. The majority of participants were from Asian countries (n = 47,511). Sixteen studies used a cross-sectional study design, and two studies used a prospective cohort study design. About 83% of the included studies used the self-report method of assessment, while the rest used the face-to-face method. The majority (14) of the studies used standardized, valid measurement instruments such as ISI, ESS, AIS, and PDSS, while the rest used self-designed questionnaires. Regarding study setting, 72% of the studies were conducted at school, and the remaining studies were conducted at household level, in the community, and in the clinical area. Sleep disturbance was reported in accordance with *ICSD-3* diagnoses [1], including insomnia, SRBDs, and hypersomnolence. We followed the grouping reported by Santoso et al. [86]. In the insomnia category, we included studies that reported insomnia, insomnia symptoms, poor sleep quality, and trouble sleeping. The hypersomnolence category included studies that reported EDS and insufficient sleep. The SRBD category included studies that reported snoring and DBS. Regarding substance use, smoking and alcohol use were reported in 15 studies, and marijuana and coffee use were reported in 3 studies. In terms of the categories, 12, 4, and 3 studies reported insomnia, hypersomnolence, and SRBDs, respectively. Figure 2 presents the proportion of sleep disturbance in relation to substance use among the studies.

**Table 1:** Demographics and characteristics of included studies

	Number of studies	Number of participants (%)	Mean	SD
Sample size	18	1,24,554		
Age	18		15	3.32
**Sex**				
Male	18	62,572 (50.2)		
Female	18	61,982 (49.8)		
**Geographic location**				
America	6	39,057 (31.4)		
Europe	6	37,665 (30.2)		
Asia	5	47,236 (37.9)		
Oceania	1	596 (0.5)		
**Study design**				
Cross-sectional	16	1,14,270 (91.7)		
Prospective cohort	2	10,284 (8.3)		
**Study setting**				
School	13	92,145 (74.0)		
Others ^a^	5	32,409 (26.0)		
**Instrument**				
Self-designed	4	58,261 (46.8)		
Others ^b^ (standardized)	14	66,293 (53.2)		
**Method of assessment**				
Self-report	15	1,00,539 (80.7)		
Face-to-face interview	3	24,015 (19.3)		
**Substance use**	**18**	**55,936 (44.9)**		
Alcohol	15	20,953 (37.5)		
Smoking	15	25,626 (45.8)		
Coffee	3	64,66 (11.5)		
Marijuana	3	2891 (5.2)		
**Sleep disturbance**				
Insomnia	12	9057 (16.2)		
Hypersomnolence	4	1625 (2.9)		
SRBD	3	5167 (9.2)		

^*a*^*= Social media, community, and clinical setting*, ^*b*^*= Adolescent Heath Questionnaire, Health Problems Checklist, Minimal Insomnia Symptom Scale, Youth Self-Report, Epworth Sleepiness Scale, Insomnia Severity Index, Pediatric Daytime Sleepiness Scale, Global Appraisal of Individual Needs-Short Screener, Athens Insomnia Scale, International Classification of Sleep Disorders-Third Edition, Diagnostic and Statistical Manual of Mental Disorders version 5, Global Sleep Assessment Questionnaire, American Academy of Sleep Medicine, Pittsburgh Sleep Quality Index, Stanford Sleep Questionnaire and Assessment of Wakefulness*, ^*c*^*= Sleep-Related Breathing Disorders*

### Study quality and risk of bias assessment

Supplementary Table S4 presents details regarding the study’s quality and risk of bias. According to the Joanna and Briggs Institute appraisal tool (JBI), all the studies used an appropriate sample to examine the targeted population, used validated outcome measurement instruments, and included an adequate sample size. Approximately 86% of the articles applied appropriate sampling of participants, and approximately 86% of the studies used appropriate statistical analysis methods. Fourteen studies had high quality and a low risk of bias, and four studies had moderate quality and a moderate risk of bias. None of the studies had low quality. The overall quality of the studies was high.

### Overall prevalence of sleep disturbance among adolescents with substance use

**Fig. 3:**
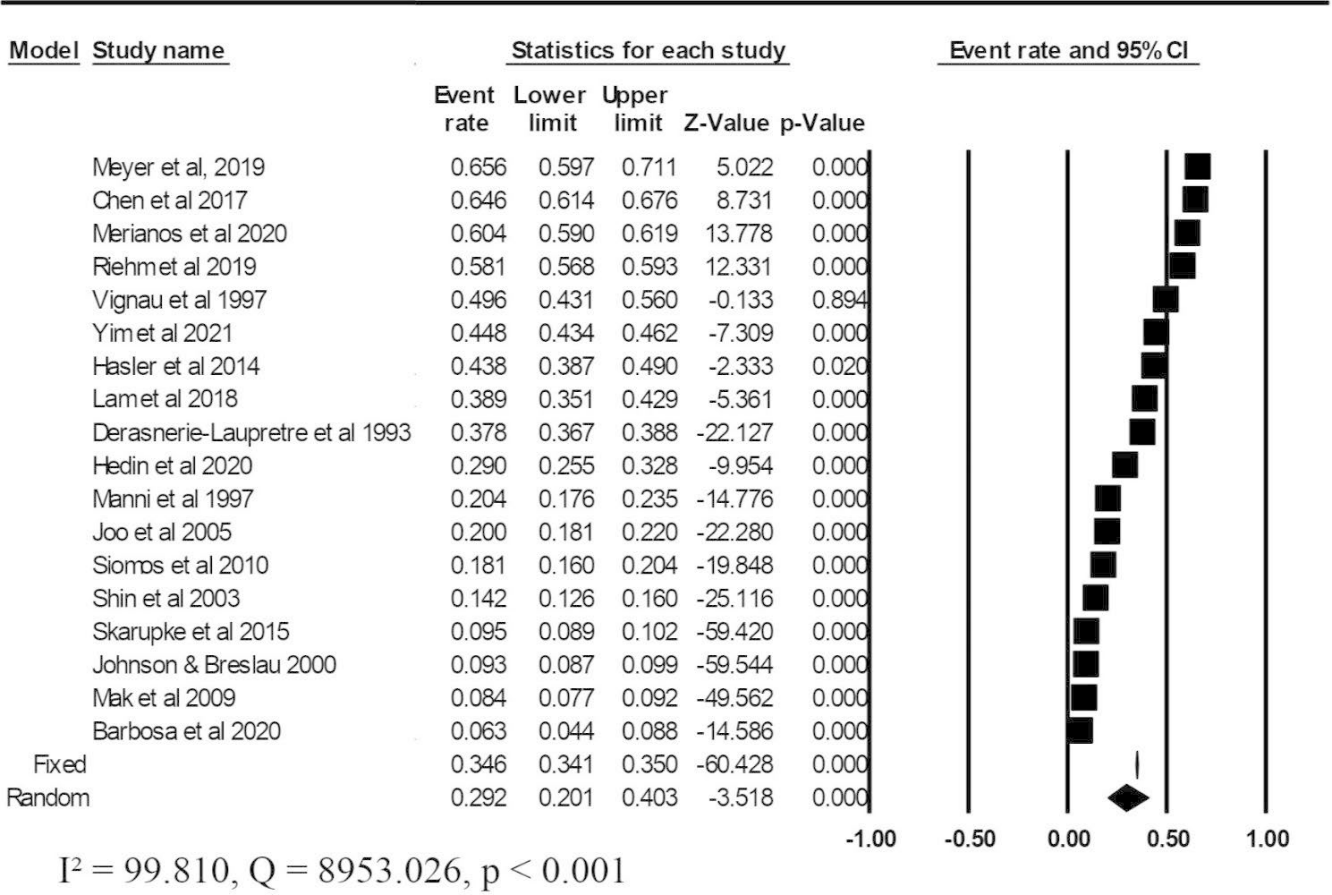
Prevalence of sleep disturbance among adolescents with substance use

Figure 3 presents the prevalence rates of sleep disturbance among adolescents with substance use. Using CMA software version 3, we pooled the prevalence rate of sleep disturbance from 18 studies, and the results indicated an overall prevalence rate of 29% (95% CI: 0.201–0.403) with heterogeneity (*I*^2^ = 99.810, *Q* statistics = 8953.026, *p* < 0.001).

#### Prevalence of sleep disturbances grouped by substance use

We further grouped the included studies by substances. The reason for the grouping was to assess the overall prevalence rate of sleep disturbances associated with alcohol, smoking, coffee, and marijuana. Table 2 presents the results of the subgroup analysis, which included all the categories of sleep disturbance grouped by substance use. The overall prevalence rate of sleep disturbance was 28% (95% CI: 0.214–0.363), with heterogeneity (*I*^2^ = 99.683, *Q* statistics = 11660.186, *p* = <0.001). Among alcohol users, coffee users, marijuana users, and smokers, the prevalence rates of sleep disturbance were 29% (95% CI: 0.175–0.445), 23% (95% CI: 082–0490), 37% (95% CI: 0.096–0.770), and 28% (95% CI: 0.191–0.392), respectively. All the groups exhibited high heterogeneity. Supplementary Figure S1 illustrates a forest plot of the analysis results.

**Table 2:** Prevalence of sleep disturbances grouped by substance use

	N Study	Prev. (%)	95% CI Lower limit	Upper limit	Heterogeneity Q value	*p* Value	*I* ^2^
Overall	38	28	0.214	0.363	11660.186	<0.001	99.683
Alcohol	15	29	0.175	0.445	4501.860	< 0.001	99.689
Coffee	3	23	0.082	0.490	252.011	< 0.001	99.206
Marijuana	3	37	0.096	0.770	741.531	< 0.001	99.819
Smoking	17	28	0.191	0.392	4991.147	< 0.001	99.679

Prev. = prevalence, CI = Confidence Interval

#### Prevalence of insomnia grouped by substance use

To know the overall prevalence rates of insomnia for each substance, we grouped articles that reported insomnia based on the substances used. We assessed insomnia with alcohol, coffee, marijuana, and smoking. Table 3 presents the findings of the subgroup analysis of the insomnia category, grouped by the substances used by adolescents. The overall prevalence rate of insomnia among adolescents with substance use was 28% (95% CI: 0.182–0.396), with high heterogeneity (*I*^2^ = 99.677, *Q* statistic = 7438.530, *p* = <0.001). The prevalence rates of insomnia among alcohol users, coffee users, marijuana users, and smokers were 31%, 23%, 26%, and 28%, respectively. All the groups had high heterogeneity. Supplementary Figure S2 presents the forest plot of the results.

**Table 3:** Prevalence of insomnia grouped by substance use

	N Study	Prev. (%)	95% CI Lower limit	Upper limit	Heterogeneity Q value	*p* Value	*I* ^2^
Overall	25	28	0.182	0.396	7438.530	<0.001	99.677
Alcohol	10	31	0.156	0.509	3340.713	< 0.001	99.731
Coffee	3	23	0.082	0.490	252.011	< 0.001	99.206
Marijuana	2	26	0.024	0.834	741.531	< 0.001	99.865
Smoking	10	28	0.135	0.487	2800.536	< 0.001	99.679

Prev. = prevalence, CI = Confidence Interval

#### Prevalence of hypersomnolence grouped by substance use

We grouped articles that reported hyphersomnolence based on the substances to assess the prevalence rates of hypersomnolence in different substances. Table 4 presents the findings of the subgroup analysis of the hypersomnolence category based on alcohol use and smoking. We did not include coffee and marijuana users because of the limited number of studies. The prevalence rate of hypersomnolence among adolescent smokers and alcohol users was 41% (95% CI: 0.230–0.610), with heterogeneity (*I*^2^ = 99.292, *Q* statistic = 847.825, *p* = <0.001). The prevalence rates of hypersomnolence among alcohol users and smokers were 31% and 46%, respectively. Both groups exhibited significantly high heterogeneity. Supplementary Figure S3 presents the forest plot of the results.

**Table 4:** Prevalence of hypersomnolence grouped by substance use

	N Study	Prev. (%)	95% CI Lower limit	Upper limit	Heterogeneity Q value	*p* Value	I^2^
Overall	7	41	0.230	0.610	847.825	<0.001	99.292
Alcohol	4	31	0.100	0.654	704.538	< 0.001	99.574
Smoking	3	46	0.232	0.700	142.257	< 0.001	98.494

*Prev. = prevalence, CI = Confidence Interval*

### Subgroup and meta-regression analyses of sleep disturbance and substance use

The subgroup and meta-regression analyses to identify the source of heterogeneity were stratified by geographical location, study setting, study design, method of assessment, and measuring instruments as categorical variables and sample size and age as continuous variables. Table 5 shows the summary of the results. Study design and methods of assessment were the significant variables for heterogeneity, with *p*-values of 0.008 and 0.048, respectively. None of the continuous variables were significant.

**Table 5:** Subgroup and Meta-regression analyses of included studies

Categorical variable	n	Prev.(%)	95% CI Lower	95% CI Upper	*p*-value
**Geographic location**					0.786
Asia	5	26	0.110	0.505	
America	6	35	0.154	0.606	
Europe	6	25	0.132	0.426	
**Study setting**					0.858
Not school	5	27	0.089	0.589	
School	13	30	0.207	0.411	
**Study design**					0.008*
Cohort studies	2	51	0.374	0.648	
Cross-sectional	16	27	0.178	0.383	
**Method of assessment**					0.048*
Face-to-face interview	2	49	0.306	0.669	
Self-reported	16	27	0.179	0.387	
**Instruments**					0.280
Self-designed	3	17	0.052	0.445	
Others (standardized)	15	32	0.208	0.458	
**Continuous variable**	**n**	**Coefficient**	**95% CI Lower**	**95% CI Upper**	***p*** **-value**
Sample size	18	-0.00	-0.000	0.000	0.333
Age	6	0.03	-0.829	0.898	0.937

^*^p = < 0.05

### Sensitivity analysis

**Fig. 4:**
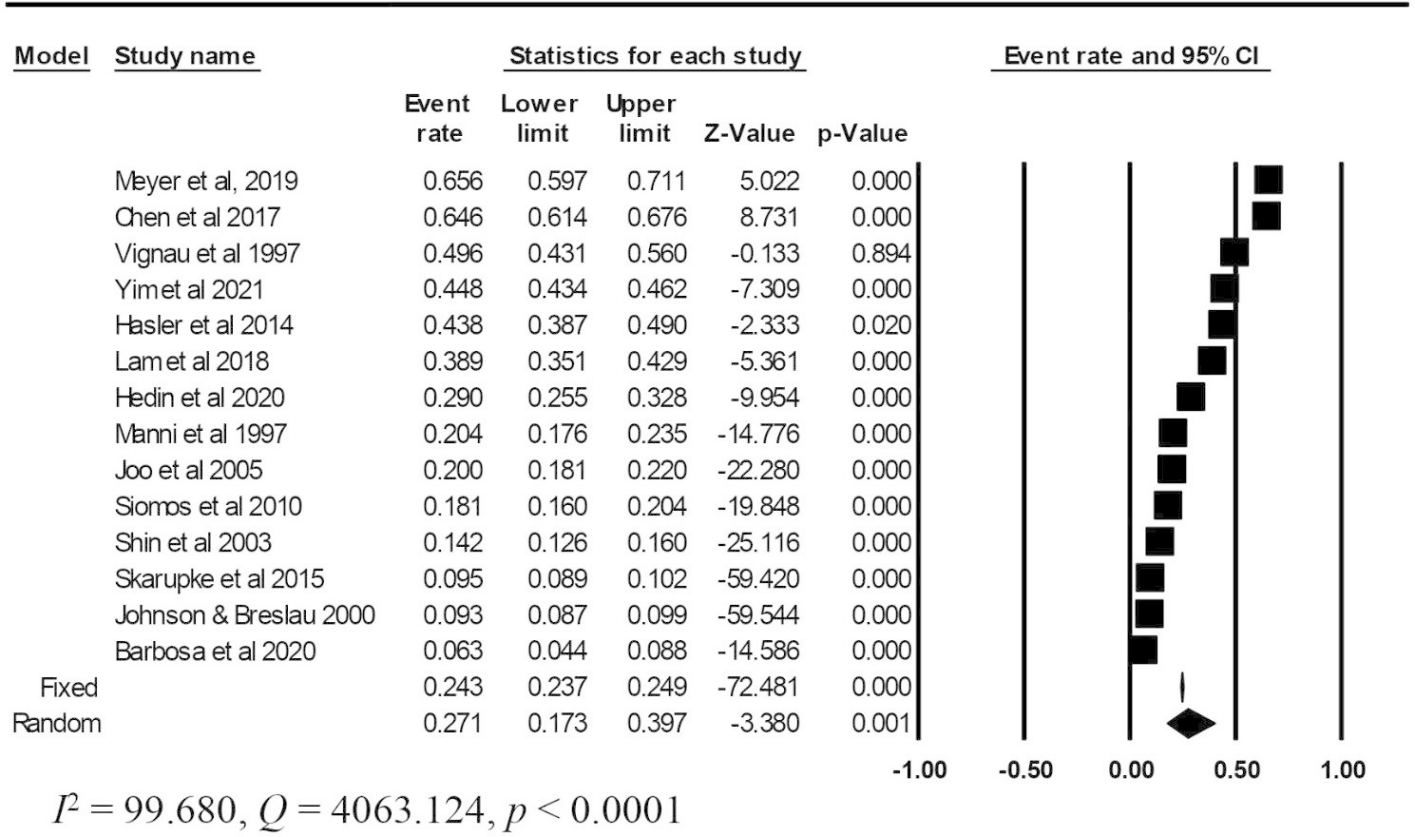
Forest plot of sensitivity analysis results obtained after the removal of large sample sizes studies

Table 6 presents the results of the sensitivity analysis. First, we used the leave-one-study-out technique. The results revealed that the overall prevalence of sleep disturbance among adolescents with substance use ranged from 27 to 31%. Then, we excluded four studies with large sample sizes [70, 76, 80, 78] The results revealed that the prevalence rate of sleep disturbance among adolescents with substance use decreased from 29 to 27% (95% CI: 0.173–0.397; Fig. 4).

**Table 6:** Sensitivity analysis performed using the leave-one-study-out technique

	Study name	Prevalence rate (%)	95% CI
1	Barbosa et al. 2020 removed	31	0.21–0.43
2	Chen et al. 2017 removed	27	0.18–0.38
3	Derasnerie-Laupretre et al. 1993 removed	29	0.18–0.41
4	Hasler et al. 2014 removed	28	0.19–0.39
5	Johnson & Breslau 2000 removed	31	0.21–0.41
6	Joo et al. 2005 removed	30	0.20–0.41
7	Lam et al. 2018 removed	29	0.19–0.40
8	Mak et al. 2009 removed	31	0.21–0.42
9	Manni et al. 1997 removed	30	0.20–0.41
10	Hedin et al. 2020 removed	29	0.19–0.40
11	Meyer et al., 2019 removed	27	0.18–0.38
12	Riehm et al. 2019 removed	28	0.18–0.38
13	Shin et al. 2003 removed	30	0.20–0.41
14	Siomos et al. 2010 removed	30	0.20–0.41
15	Skarupke et al. 2015 removed	31	0.21–0.41
16	Yim et al. 2021 removed	28	0.18–0.40
17	Vignau et al. 1997 removed	28	0.19–0.39
18	Merianos et al. 2020 removed	28	0.18–0.38

CI = Confidence interval

### Publication bias

**Fig. 5:**
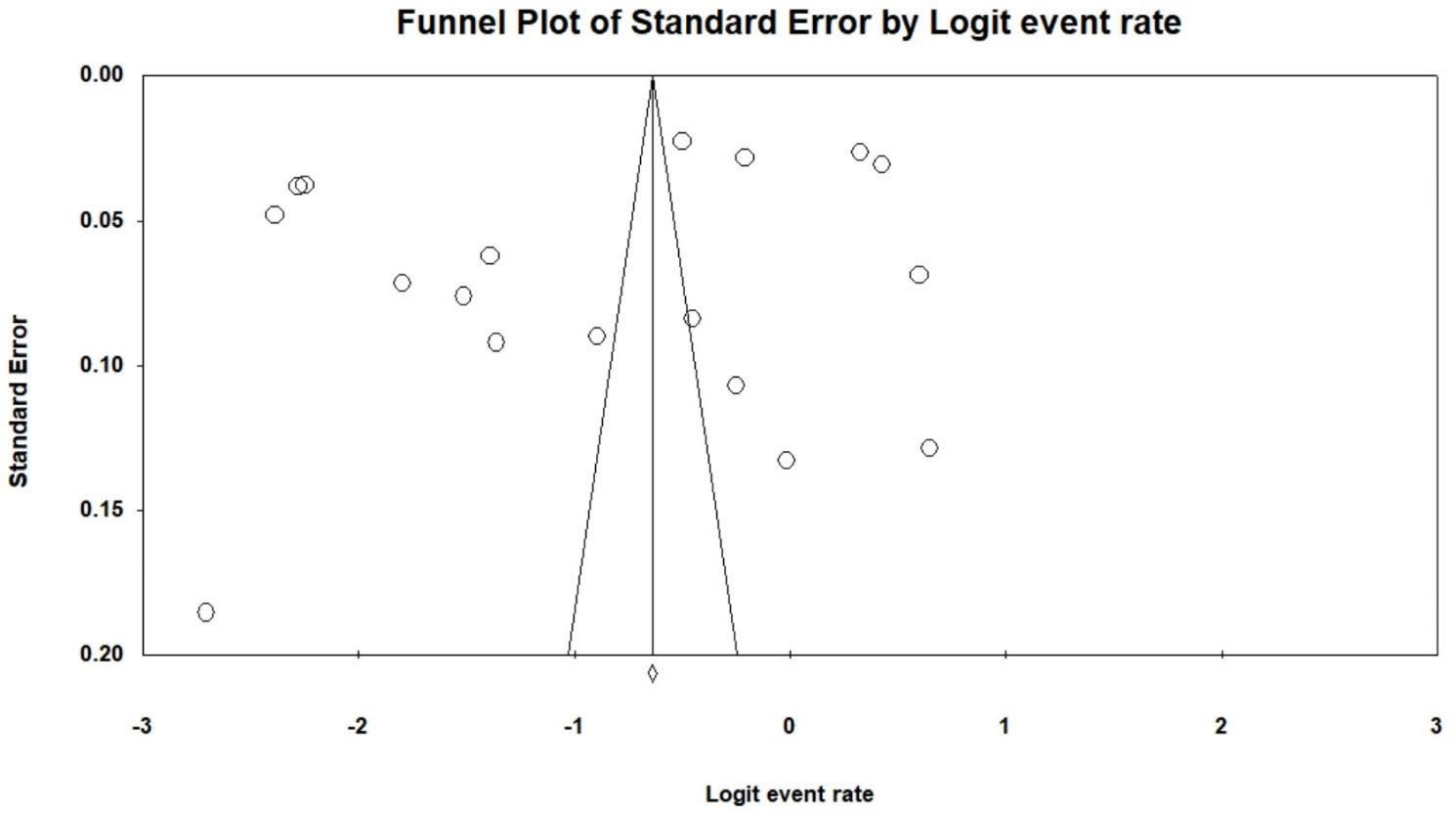
Funnel plot showing the publication bias of the included studies

Figure 5 presents the funnel plot for a visual inspection of publication bias. The results for Begg and Mazumder rank correlation and Egger’s tests [65] indicated no presence of bias and were statistically non-significant (Kendall’s Tau *b* = 0.0000, *p* = 1.000) and (*t* = 1.05227, *p* = 0.30), respectively. The Duval and Twidees’ trim and fill test revealed that no studies were trimmed.

## Discussion

This study examined the prevalence of sleep disturbance among adolescents with substance use. After pooling the prevalence rates reported in all the included studies, we determined that the overall prevalence rate of sleep disturbance among adolescents with substance use was 29%. We performed subgroup analysis of all sleep disturbance categories grouped by substances used to assess the pooled prevalence rates of sleep disturbances in each substance. The results demonstrated that adolescent marijuana users had a higher prevalence rate of sleep disturbance (37%, 95% CI: 0.096–0.770) than did adolescent alcohol users (29%), smokers (28%), and coffee users (23%; Table 2). Furthermore, we conducted a subgroup analysis of sleep disturbance categories (insomnia and hypersomnolence) to assess the prevalence rate of each sleep disturbance in different substances. The results revealed a higher prevalence rate of insomnia among alcohol users (31%; Table 3). The prevalence rate of hypersomnolence was higher among smokers than among alcohol users (46%; Table 4). Furthermore, to find the sources of heterogeneity, subgroup and meta-regression analyses were performed. The study design and method of assessment showed significant variations. Table 7 shows the overall findings of the study.

**Table 7:** Overall findings of the study

Category	Prevalence (%)	95% CI	Participants	P-value
Sleep disturbances among substance use	29	0.201–0.403	All substance users	
Sleep disturbances grouped by substance use	37	0.096–0.770	Marijuana users	
Insomnia grouped by substance use	31	0.156–0.509	Alcohol users	
Hypersomnolence grouped by substance use	46	0.232-0.700	Smokers	
**Subgroup and meta-regression**			All substance users	
Study design				0.008
Method of assessment				0.048

### Prevalence of sleep disturbance among adolescents with substance use

The overall prevalence rate of sleep disturbance among adolescents with substance use was 29% (95% CI: 0.201–0.403), ranging from 6 to 66% (Fig. 2). This rate is slightly higher than the rate of 26% (range: 8–54.7%) reported in a previous study that included Chinese adolescents without substance use [8]. This could indicate the influence of substances on sleep among adolescents. Biologically, adolescents experience sleep changes due to the influence of circadian timing and the sleep-wake cycle [87]. These changes result in a delay in both sleeping and waking time. An addition of substances such as alcohol, marijuana, coffee, and smoking, which have effects on the nervous system, would mean more sleep issues among adolescents. A previous review [88] reported a higher prevalence rate of sleep disturbance, ranging from 2 to 77%, than that observed in our study; however, the findings of the review were based on parents’ reports of sleep disturbance in their children. Thus, their finding may not reflect the actual prevalence of sleep disturbance among adolescents. Parents may record their findings on the basis of the time at which adolescents turn off bedroom lights, when they may actually still be awake and using technology devices until late hours. In the current study, we analyzed the information provided by adolescents, and the majority of them were recruited from schools. Globally, the majority of adolescents are in high and secondary schools, where both peer selection and socialization exist. According to the literature, the two-mentioned concepts have a great influence on substance use among adolescents [89]. A previous study found a decrease in substance use among adolescents during the Covid-19 pandemic lockdown [90]. This was attributed to the fact that adolescents had minimal access to substances, and the chance of using them was low due to the increased time spent with parents and guardians and the limitations of peer-group gatherings [90]. Furthermore, evidence has shown that sleep disturbance and substance use have a positive bidirectional relationship, implying a greater risk to an adolescent’s health [27]. Sleep disturbances and substance use taken together may contribute to psychological disorders such as depression and anxiety [27]. Our findings may assist health-care providers in strategizing school-targeted interventions that can be applied to a considerable number of adolescents.

#### Prevalence of sleep disturbance grouped by substance use

Our findings revealed that adolescents who used marijuana had a higher prevalence rate of sleep disturbance (37%, 95% CI: 0.096–0.770; Table 3). This rate is higher than the prevalence rate of 30% reported in a previous study, including those who used marijuana [54]. Another previous study examining college students indicated that approximately 30% of participants reported using marijuana upon registering at college, and that 8.5% reported using marijuana in their first year of college [91]. By contrast, in the current study, only 5.2% of the participants reported the use of marijuana (Table 1). Furthermore, a few (3) articles regarding marijuana use were included, which is consistent with the previous review that included only four articles [27]. Despite the fact that the percentage of marijuana use appears to be relatively low among adolescents, evidence suggests that the perception among adolescents is that marijuana is less dangerous than other substances [92]. Consequently, they frequently use it, resulting in adverse outcomes, including sleep disturbances. Marijuana has been reported to possess therapeutic effects, which led to its legalization in some countries; however, the positive effects on sleep are short-term [93, 94]. The findings of the current study have shown that sleep disturbances are prevalent among adolescents with marijuana use. The inadequate number of studies included in both the current and previous reviews may indicate a lack of an appropriate screening process for examining many adolescents in both schools and communities. More studies are needed in this area.

#### Prevalence of insomnia grouped by substance use

The subgroup analysis in our study revealed a higher prevalence rate of insomnia (31%; 95% CI: 0.156–0.509) among alcohol users (Table 3). This rate is higher than the prevalence rate of 18% reported for insomnia among alcohol users in a previous study [95]. Among adolescents with substance use in our study, 37.5% used alcohol (Table 1). Although there is evidence that consuming 2–3 drinks of alcohol may reduce the duration of sleep onset, the effects only last for a few days [96]. A previous study examined the use of alcohol as a sleep aid; however, 40% of adolescents who used alcohol to sleep developed insomnia and other sleep complaints [97]. There is evidence that alcohol alters sleep time and continuity, which are common problems among those with insomnia [98]. Our results indicated that majority of the studies used cross-sectional studies. According to the literature, most studies have used a cross-sectional study design to investigate insomnia and alcohol use, which is consistent with our findings [97]. In the previous study, the authors emphasized that individuals with insomnia should be thoroughly examined for alcohol use, as it is difficult to understand the causal relationship when a cross-sectional study design is used [97]. In general, substance use has been linked to an increase in the prevalence of insomnia [99, 100]. Similar to alcohol, marijuana was reported to decrease the duration of sleep onset latency; however, it was not clear how marijuana affects sleep quality [93]. In addition, smoking was reported to have calming effects; however, it was discovered that it disrupts sleep [101]. The link between insomnia and substance use has been reported to be bidirectional. A previous study reported that adolescents with poor sleep quality displayed a high chance of substance use later on [102]. The findings of the current study have shown that insomnia is highly prevalent and a concern that needs further research among adolescents with alcohol use. Longitudinal studies may assist in understanding more about the relationship between insomnia and alcohol.

#### Prevalence of hypersomnolence grouped by substance use

Our findings revealed that the prevalence rate of hypersomnolence was 46% (95% CI 0.232-0.700) among adolescents who smoked (Table 4). This rate is higher than the prevalence rate of 17% for hypersomnolence observed in adolescents in a previous study [26]. In the current study, 45.8% of adolescents with substance use were smokers. Nicotine present in tobacco and cigarettes affects the sleep cycle and alters sleep quality, resulting in daytime sleepiness [41]. Daytime sleepiness, or hypersomnolence affects the academic performance, thought process, and psychological health of adolescents [103, 104]. According to scientific research, parenting style may be related to adolescents’ hypersomnolence [104]. Adolescent ratings of positive parenting practices like encouragement, compliments, and support showed a strong correlation with a positive outlook on life, restorative sleep, and feelings of self-worth [104]. In addition, a good parent-adolescent relationship was found to decrease substance use among adolescents [105]. Incorporating parents in the interventions aimed at reducing hypersomnolence among smoking adolescents would be a recommendable strategy.

#### Subgroup and meta-regression analysis

Geographic location, study setting, sample size, study design, instruments, and assessment method were included in the meta-regression analysis. The subgroups of study design and method of assessment were the significant moderators, p = 0.008 and 0.048, respectively (Table 5). Our study included 18 studies, of which 16 (91.7%) used a cross-sectional study design. Although cross-sectional studies do not provide sufficient information in terms of the cause-and-effect relationship, they are preferred because they enable the examination of many variables simultaneously [106]. Regarding the method of assessment, the majority (16) of the included studies used self-reported assessment tools. The self-report assessment method is preferred because it can be applied to many individuals at the same time. In addition, it offers reliable data when all questions are answered, especially sensitive ones, reducing socially desirable bias [107, 108]. However, results, in particular, prevalence rates, may be affected due to non-response bias as some individuals may choose not to respond or unintentionally miss some questions [107]. In the current study, we found that the percentage of males was slightly higher compared to females. Gender could be a potential confounder of sleep disturbances among adolescents with substance use. Previous studies reported that despite the fact that both adolescent females and males are at risk for sleep disturbances, the likelihood of reporting sleep disturbances may be high among females, while the likelihood of using substances may be high among males [109]. Hence, gender should be considered among adolescents with substance use. Furthermore, geographical location could be a factor that affects the results of the current study. We found that the majority of the studies included in the current study were done in Asia, America, and Europe. Only one study was done in Oceania, and there was no study done in Africa. A previous review that investigated substance use in sub-Saharan Africa reported 41.6% of substance use among adolescents [110]. We would assume a high percentage of sleep disturbances among these adolescents. Future studies may consider exploring more of these locations.

### Strengths and limitations

A strength of this study is that we conducted a meta-analysis to determine the prevalence rate and categories of sleep disturbance. In addition, our study included high- to moderate-quality studies. Moreover, the majority of the included studies had adequate sample sizes. Despite these strengths, our study has some limitations that should be considered. Although the studies provided the prevalence rate of sleep disturbance, the number of studies was insufficient to pool prevalence rates among the categories of sleep disturbance and substances used. All the included studies used subjective measures of outcomes. Subjective measures provide adequate and crucial information even though they are subjected to recall bias. However, the self-reported measures of sleep disturbance provide accurate findings that are similar to those of objective measures [111, 112]. Furthermore, our study revealed high significant heterogeneity among the subgroups. However, According to the literature high heterogeneity may be unavoidable in most of the epidemiological studies [113, 114]. Another important limitation in our study is the lack of information on other psychiatric comorbidities related to sleep disturbance and substance use among adolescents. For instance, suicide thoughts, attempt and complete suicide, depression and addiction are all possible for both sleep disturbance and substance use. Understanding the related comorbidities may help in prevention and reduction strategies among adolescents. Due to these limitations, our results must be carefully interpreted. Future studies should include longitudinal studies for more insights on the effects of substance use on sleep disturbances over a longer period of time among adolescents.

### Implications for clinical practice

This study provided valuable information on factors contributing to sleep disturbance among adolescents. This information can help health-care providers develop focused effective interventions. Preventive and promotive school health educational programs can reduce susbtance use and improve regular sleep and wake timetables among adolescents.

## Conclusion

The findings of this study revealed a high prevalence of sleep disturbances among adolescents with substance use, and it was higher among marijuana users compared to alcohol users, coffee users, and smokers. In addition, high prevalence rates of insomnia and hypersomnolence were observed among alcohol users and smokers, respectively. Furthermore, study design and method of assessment were significant moderators of sleep disturbances among adolescents with substance use. Due to limitations in the number of studies, the use of a variety of self-reported assessment tools, the high heterogeneity among the groups, and the lack of information on other comorbidities, the findings must be interpreted with caution. The results indicate that sleep disturbances among adolescents with substance use could be a global concern that warrants research attention. Strategizing effective targeted interventions that can reduce substance use, prevent sleep disturbance, and promote healthy sleep habits among adolescents would be necessary. Future studies may consider longitudinal studies to evaluate the effects of substance use on sleep disturbances over a longer period time.

### Electronic supplementary material

Below is the link to the electronic supplementary material.

Supplementary Figure S1. Prevalence of sleep disturbances grouped by substance use.

Supplementary Figure S2. Prevalence of insomnia grouped by substance use.

Supplementary Figure S3. Prevalence of hypersomnolence grouped by substance use.

Supplementary Tables. S1-S4. Assessment of the quality of the studies and the risk of bias

## Data Availability

The data used and/or analyzed as part of the present study are available from the corresponding author on reasonable request.
