# Supplementary figures and images for: Prevalence of sleep disturbance among adolescents with substance use: a systematic review and meta-analysis
Source: Child Adolesc Psychiatry Ment Health. 2023 Aug 26;17:100. doi: 10.1186/s13034-023-00644-5 (PMC10464186; doi:10.1186/s13034-023-00644-5)

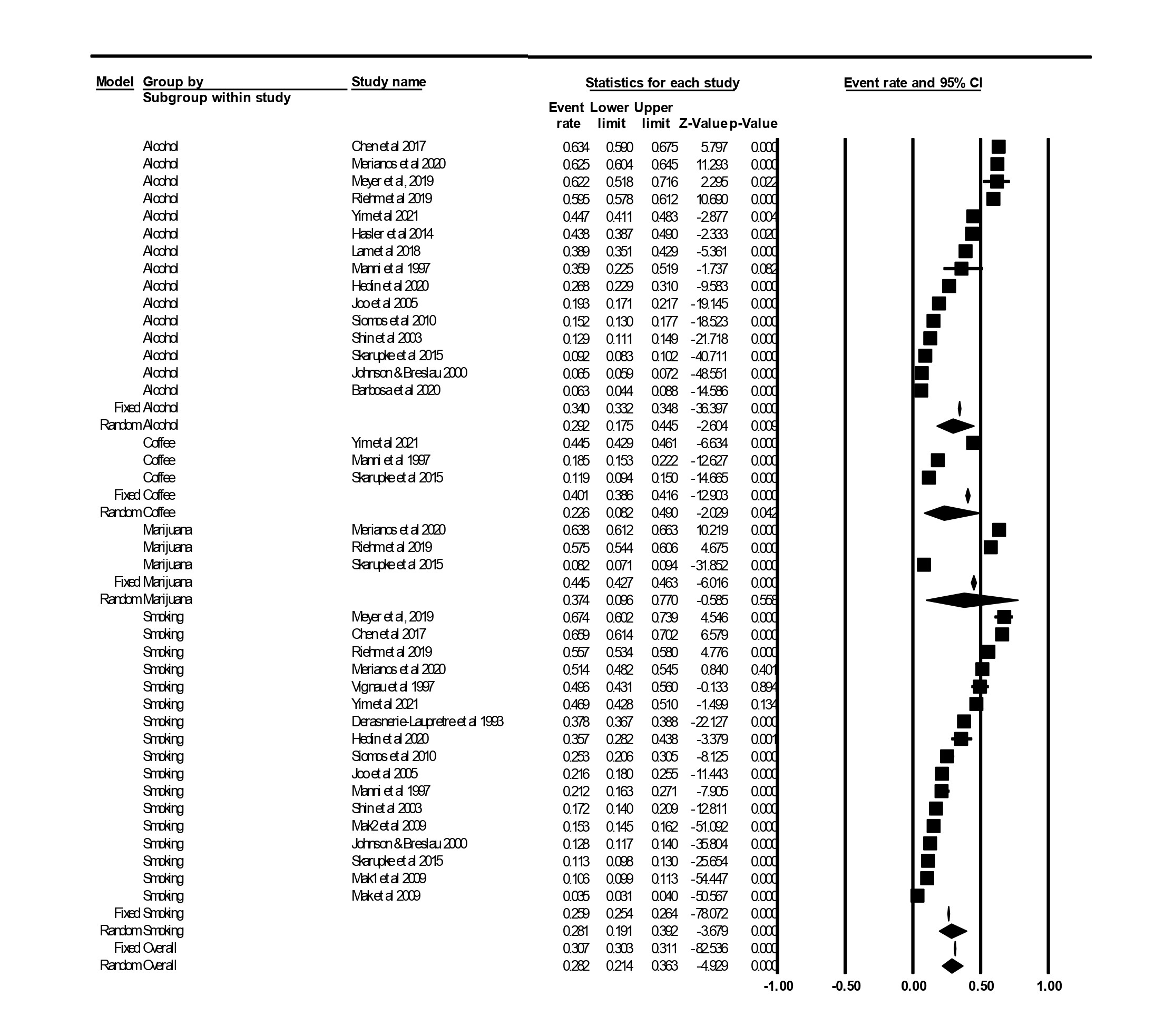

Supplement: Supplementary file 1 — Supplementary Figure S1. Prevalence of sleep disturbances grouped by substance use. [file 13034_2023_644_MOESM1_ESM.jpg]

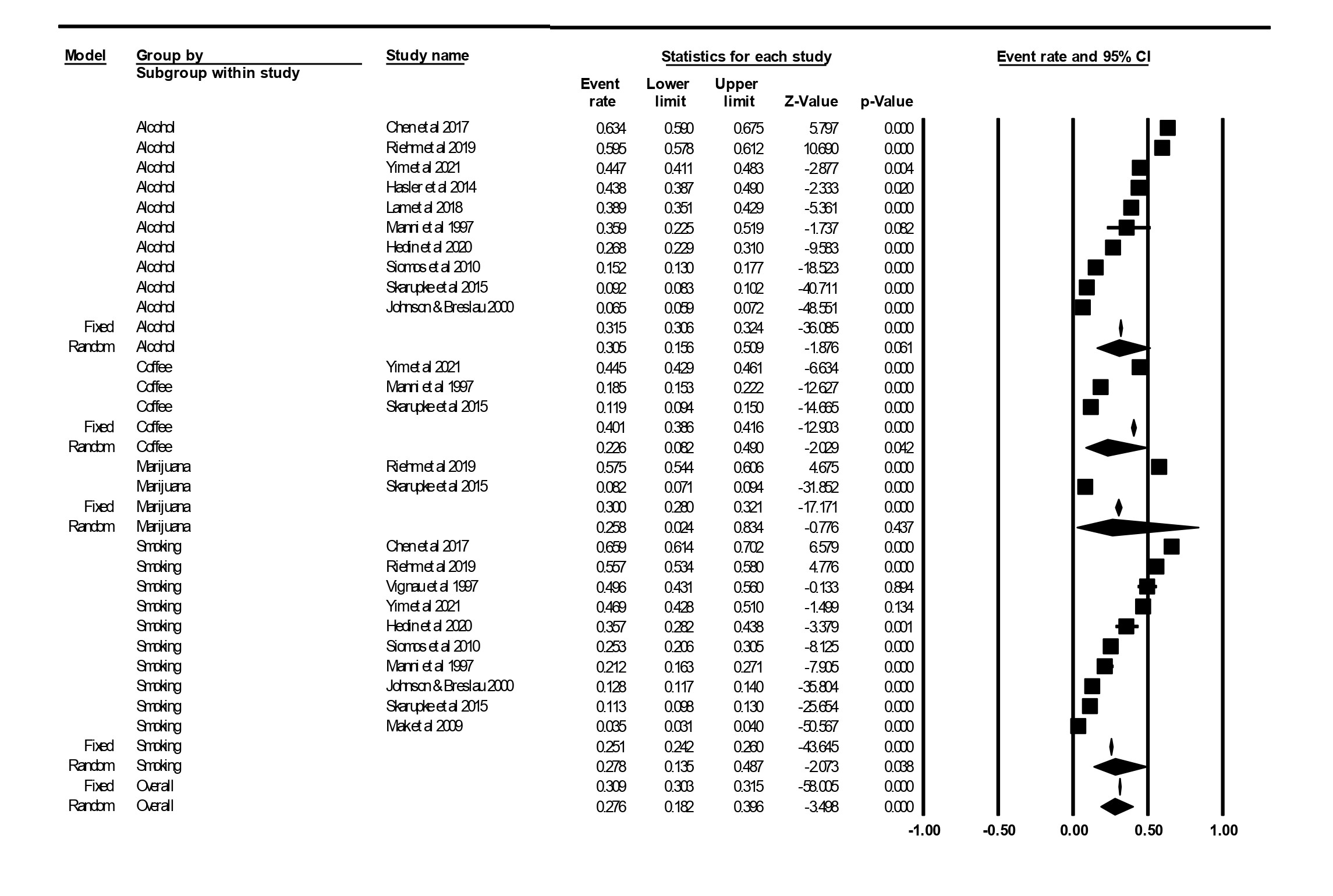

Supplement: Supplementary file 2 — Supplementary Figure S2. Prevalence of insomnia grouped by substance use. [file 13034_2023_644_MOESM2_ESM.jpg]

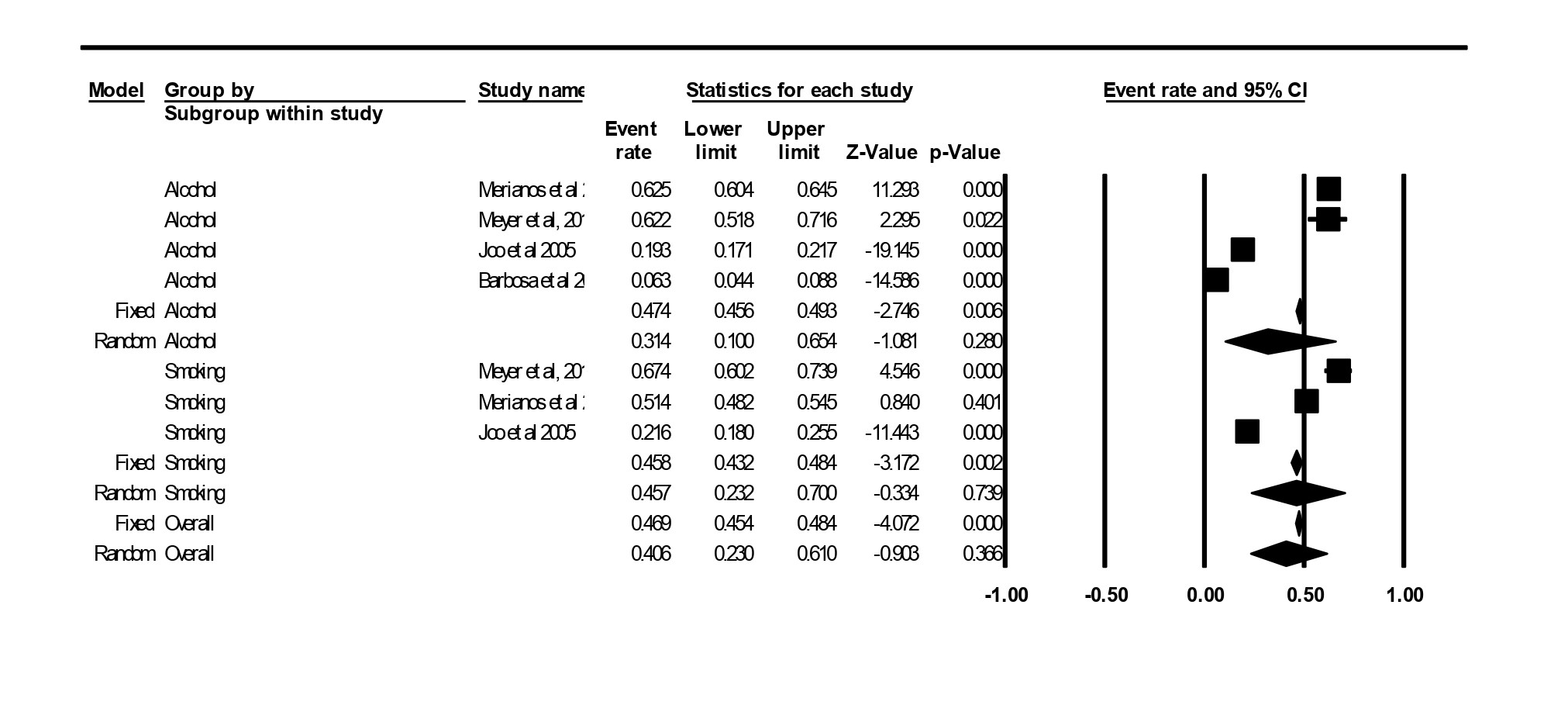

Supplement: Supplementary file 3 — Supplementary Figure S3. Prevalence of hypersomnolence grouped by substance use. [file 13034_2023_644_MOESM3_ESM.jpg]
