# Supplementary material for: Prevalence of sleep disturbance among adolescents with substance use: a systematic review and meta-analysis
Source: Child Adolesc Psychiatry Ment Health. 2023 Aug 26;17:100. doi: 10.1186/s13034-023-00644-5 (PMC10464186; doi:10.1186/s13034-023-00644-5)
Supplement: Supplementary file 4 — Supplementary Tables. S1-S4. Assessment of the quality of the studies and the risk of bias [file 13034_2023_644_MOESM4_ESM.docx]

Supplementary Table S1: Search strategy

| **Database** | **#** |  | **Search terms** |
| --- | --- | --- | --- |
| **Embase** | **1** | **P (F)** | (Teenage* OR adolescent): ti,ab,kw,de |
|  | **2** | **P (C)** | “Adolescence”/exp OR “teenager”/exp |
|  | **3** | **E (F)** | (“Substance use” OR “alcohol use” OR “marijuana use” OR “tobacco smoking” OR “cannabis use” OR “coffee use” OR “cigarette smoking”): ti,ab,kw,de |
|  | **4** | **E(C)** | “Substance-related disorders”/exp OR “alcohol drinking”/exp OR “marijuana use”/exp OR “tobacco smoking”/exp OR cannabis/exp OR coffee OR “cigarette smoking”/exp |
|  | **5** | **O(F)** | (“Sleep disturbances” OR insomnia OR “inadequate sleep” OR sleeplessness OR Sleep OR hypersomnolence OR  “Sleep problem” OR “sleep-related breathing disorders” OR “insufficient sleep” OR): ti,ab,kw,de |
|  | **6** | **O(C)** | “Sleep initiation and maintenance disorders”/exp OR “disorders of excessive somnolence”/exp OR “sleep apnea syndromes” |
|  | **7** | **All** | (#1 OR #2) AND (#3 OR #4) AND (#5 OR #6) |
|  |  |  |  |
| **PubMed** | **1** | **P(F)** | ((Adolescent[tw]) OR teenager [tw]) |
|  | **2** | **P(C)** | (Adolescence[mh] OR teenager [mh]) |
|  | **3** | **E(F)** | (((((((“Substance-related disorders” [tw]) OR “alcohol use”[tw]) OR “marijuana use”[tw]) OR “coffee use”[tw]) OR “tobacco smoking”[tw]) OR “cannabis use”[tw]) OR “cigarette smoking”[tw]) |
|  | **4** | **E(C)** | (“Substance-related disorders”[mh] OR “alcohol drinking”[mh] OR “marijuana use” [mh] OR “tobacco smoking”[mh] OR cannabis[mh] OR coffee[mh] OR “cigarette smoking”[mh]) |
|  | **5** | **O(F)** | (((((((((“Sleep disturbances”[tw]) OR insomnia[tw]) OR “inadequate sleep”[tw]) OR sleeplessness[tw]) OR sleep[tw]) OR hypersomnolence[tw]) OR “sleep problems”[tw]) OR “sleep-related breathing disorders”[tw]) OR “insufficient sleep”[tw]) |
|  | **6** | **O(C)** | (“sleep initiation and maintenance disorders”[mh] OR “disorders of excessive somnolence”[mh] OR “sleep apnea syndromes”[mh]) |
|  | **7** | **All** | (#1 OR #2) AND (#2 OR #3) AND (#5 OR #6) |
|  |  |  |  |
| **Medline(ovid)** | **1** | **P(F)** | (Teenager OR adolescent).mp |
|  | **2** | **P(C)** | Exp Adolescence/ OR exp teenager/ |
|  | **3** | **E(F)** | (“substance-related disorders” OR “alcohol use” OR “marijuana use” OR “Coffee use” OR “tobacco smoking” OR “cannabis use” OR “cigarette smoking”).mp |
|  | **4** | **E(C)** | Exp “Substance-related disorders”/ OR exp “alcohol drinking”/ OR exp “marijuana use”/ OR exp “cannabis”/ OR exp coffee/ OR exp “cigarette smoking”/ OR exp “tobacco smoking”/ |
|  | **5** | **O(F)** | (“Sleep disturbances” OR insomnia OR “inadequate sleep” OR sleeplessness OR sleep OR hypersomnolence OR “sleep problems” OR “Sleep-related breathing disorders” OR “insufficient sleep”).mp |
|  | **6** | **O(C)** | Exp “sleep initiation and maintenance disorders”/ OR exp “disorders of excessive somnolence”/ OR exp “disorders of excessive somnolence”/ OR exp “sleep apnea syndromes”/ |
|  | **7** | **All** | (#1 OR # 2) AND (#3 OR #4) AND (#5 OR #6) |
|  |  |  |  |
| **CINHAL** | **1** | **P(F)** | Adolescent OR teenager |
|  | **2** | **P(C)** | (Adolescence) OR (teenager) |
|  | **3** | **E(F)** | Substance-related disorders OR alcohol use OR marijuana use OR coffee use OR tobacco smoking OR cannabis use OR cigarette smoking. |
|  | **4** | **E(C)** | (Substance-related disorders) OR (alcohol drinking) OR (marijuana use) OR (tobacco smoking) OR (cigarette smoking) OR coffee OR cannabis |
|  | **5** | **O(F)** | Sleep disturbances OR insomnia OR inadequate sleep OR sleeplessness OR sleep OR hypersomnolence OR sleep problems OR sleep-related breathing disorders OR insufficient sleep |
|  | **6** | **O(C)** | (Sleep initiation and maintenance disorders) OR (disorders of excessive hypersomnolence) OR (sleep apnea syndromes) |
|  | **7** | **All** | (#1 OR # 2) AND (#3 OR #4) AND (#5 OR #6) |
|  |  |  |  |
| **WoS** | **1** | **P(F)** | Ts = adolescent OR teenager |
|  | **2** | **P(C)** | Ts = adolescent OR teenager |
|  | **3** | **E(F)** | Ts = substance-related disorders OR alcohol use OR marijuana use OR coffee use OR tobacco smoking OR cannabis use OR cigarette smoking |
|  | **4** | **E(C)** | Ts = substance-related disorders OR alcohol drinking OR marijuana use OR tobacco smoking OR cigarette smoking OR cannabis OR coffee |
|  | **5** | **O (F)** | Ts = Sleep disturbances OR insomnia OR inadequate sleep OR sleeplessness OR sleep OR hypersomnolence OR sleep problems OR sleep-related breathing disorders OR insufficient sleep |
|  | **6** | **O(C)** | Ts = Sleep initiation and maintenance disorders OR Disorders of excessive hypersomnolence OR Sleep apnea syndrome |
|  | **7** | **All** | (#1 OR # 2) AND (#3 OR #4) AND (#5 OR #6) AND (#7) |
|  |  |  |  |
| **Scopus** | **1** | **P(F)** | TITLE-ABS-KEY (adolescent OR teenager) |
|  | **2** | **P(C)** | TITLE-ABS-KEY (adolescence OR teenager) |
|  | **3** | **E(F)** | TITLE-ABS-KEY (“substance-related disorders” OR “alcohol use” OR “marijuana use” OR “coffee use” OR “tobacco smoking” OR “cannabis use” OR “cigarette smoking”). |
|  | **4** | **E(C)** | TITLE-ABS-KEY (“Substance-related disorders” OR “alcohol drinking” OR “Marijuana use” OR “tobacco smoking” OR “cigarette smoking” OR “cannabis” OR “coffee”) |
|  | **5** | **O(F)** | TITLE-ABS-KEY (“Sleep disturbances” OR insomnia OR “inadequate sleep” OR sleeplessness OR Sleep OR hypersomnolence OR sleep problems OR “sleep-related breathing disorders” OR “insufficient sleep”) |
|  | **6** | **O(C)** | TITLE-ABS-KEY (“Sleep initiation and maintenance disorders” OR “disorders of excessive hypersomnolence” OR “sleep apnea syndromes”) |
|  | **7** | **All** | (#1) AND (# 2) AND (#3) AND (#4) |
|  |  |  |  |
| **ProQuest** | **1** | **P(F)** | Adolescent OR teenager |
|  | **2** | **P(C)** | Adolescence OR teenager |
|  | **3** | **E(F)** | Substance-related disorders OR alcohol use OR marijuana use OR coffee use OR tobacco smoking OR cannabis use OR cigarette smoking. |
|  | **4** | **E(C)** | Substance-related disorders OR alcohol drinking OR marijuana use OR tobacco smoking OR cigarette smoking OR coffee OR cannabis |
|  | **5** | **O(F)** | Sleep disturbances OR insomnia OR inadequate sleep OR sleeplessness OR sleep OR hypersomnolence OR sleep problems OR sleep-related breathing disorders OR insufficient sleep |
|  | **6** | **O(C)** | Sleep initiation and maintenance disorders OR disorders of excessive hypersomnolence OR sleep apnea syndromes |
|  | **7** | **All** | (#1 OR # 2) AND (#3 OR #4) AND (#5 OR #6) |

*P = population, E = exposure, O = outcome, C = control text (MeSH terms), F = free text*

Supplementary Table S2: List of included studies

| **Number** | **Study** |
| --- | --- |
| 1 | Barbosa, S., Batista, R. F. L., Rodrigues, L. D. S., Braganca, M., Oliveira, B. R., Simoes, V. M. F., . . . Silva, A. Prevalence of excessive daytime sleepiness and associated factors in adolescents of the RPS cohort, in Sao Luis (MA), Brazil. Revista Brasileira de Epidemiologia, 23, e200071. |
| 2 | Chen, H., Bo, Q.-G., Jia, C.-X., & Liu, X. (2017). Sleep problems in relation to smoking and alcohol use in Chinese adolescents. *The Journal of nervous and mental disease, 205*(5), 353-360. |
| 3 | Delasnerie‐Laupretre, n., Patois, e., Valatx, j. L., Kauffmann, f., & Alperovitch, a. (1993). Sleep, snoring and smoking in high school students. *Journal of Sleep Research, 2*(3), 138-142. |
| 4 | Hasler, B. P., Martin, C. S., Wood, D. S., Rosario, B., & Clark, D. B. (2014). A Longitudinal Study of Insomnia and Other Sleep Complaints in Adolescents With and Without Alcohol Use Disorders. *Alcoholism: Clinical & Experimental Research, 38*(8), 2225-2233. doi:10.1111/acer.12474 |
| 5 | Hedin, G., Norell-Clarke, A., Hagell, P., Tonnesen, H., Westergren, A., & Garmy, P. (2020). Insomnia in Relation to Academic Performance, Self-Reported Health, Physical Activity, and Substance Use Among Adolescents. *International Journal of Environmental Research & Public Health [Electronic Resource], 17*(17), 03. |
| 6 | Johnson, E. O., & Breslau, N. (2001). Sleep problems and substance use in adolescence. *Drug and Alcohol Dependence, 64*(1), 1-7. |
| 7 | Joo, S., Shin, C., Kim, J., Yi, H., Ahn, Y., Park, M., . . . Lee, S. (2005). Prevalence and correlates of excessive daytime sleepiness in high school students in Korea. *Psychiatry and Clinical Neurosciences, 59*(Sunderland et al.), 433-440. |
| 8 | Lam, T., Ogeil, R. P., Allsop, S., Chikritzhs, T., Fischer, J., Midford, R., . . . Lloyd, B. (2018). Insomnia and regulation of sleep-wake cycle with drugs among adolescent risky drinkers. *Journal of Clinical Sleep Medicine, 14*(9), 1529-1537. |
| 9 | Mak, K.-K., Ho, S.-Y., Thomas, G. N., Lo, W.-S., Cheuk, D. K.-L., Lai, Y.-K., & Lam, T.-H. (2010). Smoking and sleep disorders in Chinese adolescents. *Sleep Medicine, 11*(3), 268-273. doi:https://doi.org/10.1016/j.sleep.2009.07.017 |
| 10 | Manni, R., Ratti, M. T., Marchioni, E., Castelnovo, G., Murelli, R., Sartori, I., . . . Tartara, A. (1997). Poor sleep in adolescents: a study of 869 17-year-old Italian secondary school students. *J Sleep Res, 6*(1), 44-49. doi:10.1046/j.1365-2869.1997.00025.x |
| 11 | Merianos, A. L., Jandarov, R. A., Choi, K., Fiser, K. A., & Mahabee-Gittens, E. M. (2021). Combustible and electronic cigarette use and insufficient sleep among US high school students. *Preventive Medicine, 147*, 106505. |
| 12 | Meyer, C., Ferrari Junior, G. J., Andrade, R. D., Barbosa, D. G., da Silva, R. C., Pelegrini, A., & Gomes Felden, É. P. (2019). Factors associated with excessive daytime sleepiness among Brazilian adolescents. *Chronobiology International, 36*(9), 1240-1248. doi:10.1080/07420528.2019.1633661 |
| 13 | Riehm, K. E., Rojo-Wissar, D. M., Feder, K. A., Mojtabai, R., Spira, A. P., Thrul, J., & Crum, R. M. (2019). E-cigarette use and sleep-related complaints among youth. *Journal of Adolescence, 76*, 48-54. doi:https://doi.org/10.1016/j.adolescence.2019.08.009 |
| 14 | Shin, C., Joo, S., Kim, J., & Kim, T. (2003). Prevalence and Correlates of Habitual Snoring in High School Students*. *CHEST, 124*(5), 1709-1715. doi:https://doi.org/10.1378/chest.124.5.1709 |
| 15 | Siomos, K. E., Avagianou, P.-A., Floros, G. D., Skenteris, N., Mouzas, O. D., Theodorou, K., & Angelopoulos, N. V. (2010). Psychosocial correlates of insomnia in an adolescent population. *Child Psychiatry and Human Development, 41*(3), 262-273. doi:10.1007/s10578-009-0166-5 |
| 16 | Skarupke, C., Schlack, R., Lange, K., Goerke, M., Dueck, A., Thome, J., . . . Cohrs, S. (2017). Insomnia complaints and substance use in German adolescents: did we underestimate the role of coffee consumption? Results of the KiGGS study. *J Neural Transm (Vienna), 124*(Suppl 1), 69-78. doi:10.1007/s00702-015-1448-7 |
| 17 | Vignau, J., Bailly, D., Duhamel, A., Vervaecke, P., Beuscart, R., & Collinet, C. (1997). Epidemiologic study of sleep quality and troubles in French secondary school adolescents. *Journal of Adolescent Health, 21*(5), 343-350. |
| 18 | Yim, S. H., Yang, K. I., Kim, J. H., Hwangbo, Y., Kim, D., & Hong, S. B. (2021). Association between eveningness preference, socio-behavioral factors, and insomnia symptoms in Korean adolescents. *Sleep Medicine, 82*, 144-150. doi:https://doi.org/10.1016/j.sleep.2021.03.016 |

Supplementary Table S3: Characteristics of included studies

|  | **Author/year** | **Country/location** | **Age (mean/median) (years)** | **Number of women (%)** | **Study sesign** | **Study Setting** | **Substance** | **Sample size** | **Sleep disturbance** | **Instrument** | **Assessment method** |
| --- | --- | --- | --- | --- | --- | --- | --- | --- | --- | --- | --- |
| 1 | Barbosa et al 2020 | Brazil, South America | 18 to 19 | 52 | Cross sectional | School | Alcohol | 2514 | EDS | ESS | Self  -report |
| 2 | Chen et al 2017 | China, Asia | (15.5, SD = 2.1) | 51 | Cross sectional | School | Alcohol  Smoking | 2090 | Poor sleep quality | AHQ | Self-report |
| 3 | Derasnerie-Laupretre et al 1993 | France, Europe | 15–20 (17.3, SD = 2.3) | 54 | Cross sectional | School | smoking | 24682 | Snoring | Self-designed questionnaire | Self-report |
| 4 | Hasler et al 2014 | USA, North America | 12–19 | 47 | Prospective cohort | Clinical,  Community | Alcohol | 696 | Insomnia | HPC | Self-report |
| 5 | Hedin et al 2020 | Sweden, Europe | 15–17 (16) | 56 | Cross sectional | School | Alcohol  Smoking | 1477 | Insomnia | MISS | Self-report |
| 6 | Johnson & Breslau 2000 | USA, North America | 12–17 | 49 | Cross sectional | Household | Alcohol  Smoking | 13831 | Trouble sleeping | YSR checklist | Face-to-face interview |
| 7 | Joo et al 2005 | South Korea, Asia | 15–18 (F = 16.9, M = 16.8) | 30 | Cross sectional | School | Alcohol  Smoking | 3871 | EDS | ESS | Self-report |
| 8 | Lam et al 2018 | Australia, Oceania | 14–19 (17.2) | 48 | Cross sectional | Social media | Alcohol | 596 | Insomnia | ISI | Face-to-face interview |
| 9 | Mak et al 2009 | Hong Kong, Asia | 12–18 | 50 | Cross sectional | School | Smoking | 28839 | DBS, Snoring, Insomnia | Self-designed questionnaire | Self-report |
| 10 | Manni et al 1997 | Italy, Europe | 17 | 61 | Cross sectional | School | Alcohol  Smoking  Coffee | 869 | Poor sleep quality | Self-designed questionnaire | Self-report |
| 11 | Merianos et al 2020 | USA, North America | 13–18 | 54 | Cross sectional | School | Alcohol  Smoking  Marijuana | 11296 | Insufficient sleep | AASM | Self-report |
| 12 | Meyer et al, 2019 | Brazil, South America | 14–19 (16.1, SD = 1.1) | 54 | Cross sectional | School | Alcohol  Smoking | 1132 | EDS | PDSS | Self-report |
| 13 | Riehm et al 2019 | USA, North America | 12–17 | 49 | Prospective Cohort | Household | Alcohol  Smoking  Marijuana | 9588 | Sleep trouble | GAIN-SS | Face-to-face interview |
| 14 | Shin et al 2003 | South Korea, Asia | 15–18 (F = 16.9, M = 16.8) | 30 | Cross sectional | School | Alcohol  Smoking | 3871 | Snoring | Self-designed questionnaire | Self-report |
| 15 | Siomos et al 2010 | Greece, Europe | (F = 16.9, M = 16.8) | 51 | Cross sectional | School | Alcohol  Smoking | 2195 | Insomnia | AIS | Self-report |
| 16 | Skarupke et al 2015 | German, Europe | 11–17 years | 49 | Cross sectional | Community | Alcohol  Smoking  Marijuana  Coffee | 7698 | Insomnia | ICSD-2, DSM-5 | Self-report |
| 17 | Yim et al 2021 | South Korea, Asia | (16.77) SD = 085) | 48 | Cross sectional | School | Alcohol  Smoking  Coffee | 8565 | Insomnia | GSAQ | Self-report |
| 18 | Vignau et al 1997 | France, Europe | 15–23 (17.2, SD = 1.5) | 41 | Cross sectional | School | Smoking | 744 | Poor sleep quality | INSERM | Self-report |

SD = Standard deviation, EDS = Excessive Daytime Sleepiness, AHQ = Adolescent Heath Questionnaire, HPC = Health Problem Checklist, MISS = Minimal Insomnia Symptom Scale, YSR = Youth Self-Report, ESS = Epworth Sleepiness Scale, ISI = Insomnia Severity Index, PDSS = Pediatric Daytime Sleepiness Scale, GAINSS = Global Appraisal of Individual Needs-Short Screener, AIS =Athens Insomnia Scale, ICSD-2= International Classification of Sleep Disorders (version 2), DSM-5=Diagnostic and Statistical Manual of Mental Disorders (version 5), GSAQ = Global Sleep Assessment Questionnaire, AASM = American Academy of Sleep Medicine, PSQI = Pittsburgh Sleep Quality Index, SSQAW = Stanford Sleep Questionnaire and Assessment of Wakefulness

Supplementary Table S4: Study quality assessment and risk of bias

| **No** | **Author/year** | **Q1** | **Q2** | **Q3** | **Q4** | **Q5** | **Q6** | **Q7** | **Q8** | **Q9** | **Total positive score** |
| --- | --- | --- | --- | --- | --- | --- | --- | --- | --- | --- | --- |
| 1 | Barbosa et al 2020 | Y | Y | Y | Y | Y | Y | Y | Y | Y | **9** |
| 2 | Chen et al 2017 | Y | Y | Y | Y | Y | Y | Y | N | Y | **8** |
| 3 | Derasnerie-Laupretre et al 1993 | Y | Y | Y | Y | Y | Y | Unclear | N | Y | **6** |
| 4 | Hasler et al 2014 | Y | N | Y | Y | Y | Y | N | NA | Y | **6** |
| 5 | Hedin et al 2020 | Y | N | Y | Y | Y | Y | N | Y | Y | **7** |
| 6 | Johnson & Breslau 2000 | Y | Y | Y | Y | Y | Y | Unclear | Y | NA | **7** |
| 7 | Joo et al 2005 | Y | Y | Y | Y | N | Y | Unclear | Y | Y | **7** |
| 8 | Lam et al 2018 | Y | N | Y | Y | Y | Y | N | Y | N | **5** |
| 9 | Mak et al 2009 | Y | Y | Y | Y | Y | Y | NA | Y | Y | **8** |
| 10 | Manni et al 1997 | Y | Y | Y | Y | Unclear | Y | NA | Y | Y | **7** |
| 11 | Meyer et al, 2019 | Y | Y | Y | Y | Y | Y | Y | Y | Unclear | **8** |
| 12 | Riehm et al 2019 | Y | Y | Y | Y | Y | Y | Unclear | Y | Y | **8** |
| 13 | Shin et al 2003 | Y | Y | Y | Y | Unclear | Y | N | Y | Y | **7** |
| 14 | Siomos et al 2010 | Y | Y | Y | Y | Unclear | Y | N | Y | Y | **7** |
| 15 | Skarupke et al 2015 | Y | Y | Y | Y | N | Y | N | Y | Unclear | **6** |
| 16 | Yim et al 2021 | Y | Y | Y | Y | Unclear | Y | N | Y | Y | **7** |
| 17 | Vignau et al 1997 | Y | Y | Y | Y | Y | Y | Y | Y | Y | **9** |
| 18 | Merianos et al 2020 | Y | Y | Y | Y | Y | Y | Y | Y | Y | **9** |

1. Q1–9: Questions to assess study quality and risk of bias, as listed below

Q1: Was the sample frame appropriate to address the target population?

Q2: Were study participants sampled in an appropriate manner?

Q3: Was the sample size adequate?

Q4: Were the study subjects and the setting described in detail?

Q5: Was the data analysis conducted with sufficient coverage of the identified sample?

Q6: Were valid methods used for the identification of the condition?

Q7: Was the condition measured in a standard and reliable manner for all participants?

Q8: Was appropriate statistical analysis conducted?

Q9: Was the response rate adequate, and if not, was the low response rate managed appropriately?

2. Y = yes, N = no; U = unclear; NA = not applicable.
